# Supplementary material for: Healthful Eating Behaviors among Couples Contribute to Lower Gestational Weight Gain
Source: Nutrients. 2024 Mar 13;16(6):822. doi: 10.3390/nu16060822 (PMC10974170; doi:10.3390/nu16060822)
Supplement: Supplementary file 1 [file nutrients-16-00822-s001.zip › nutrients-2857337-supplementary.pdf]

Figure S1. Analytic Sample Flow Diagram

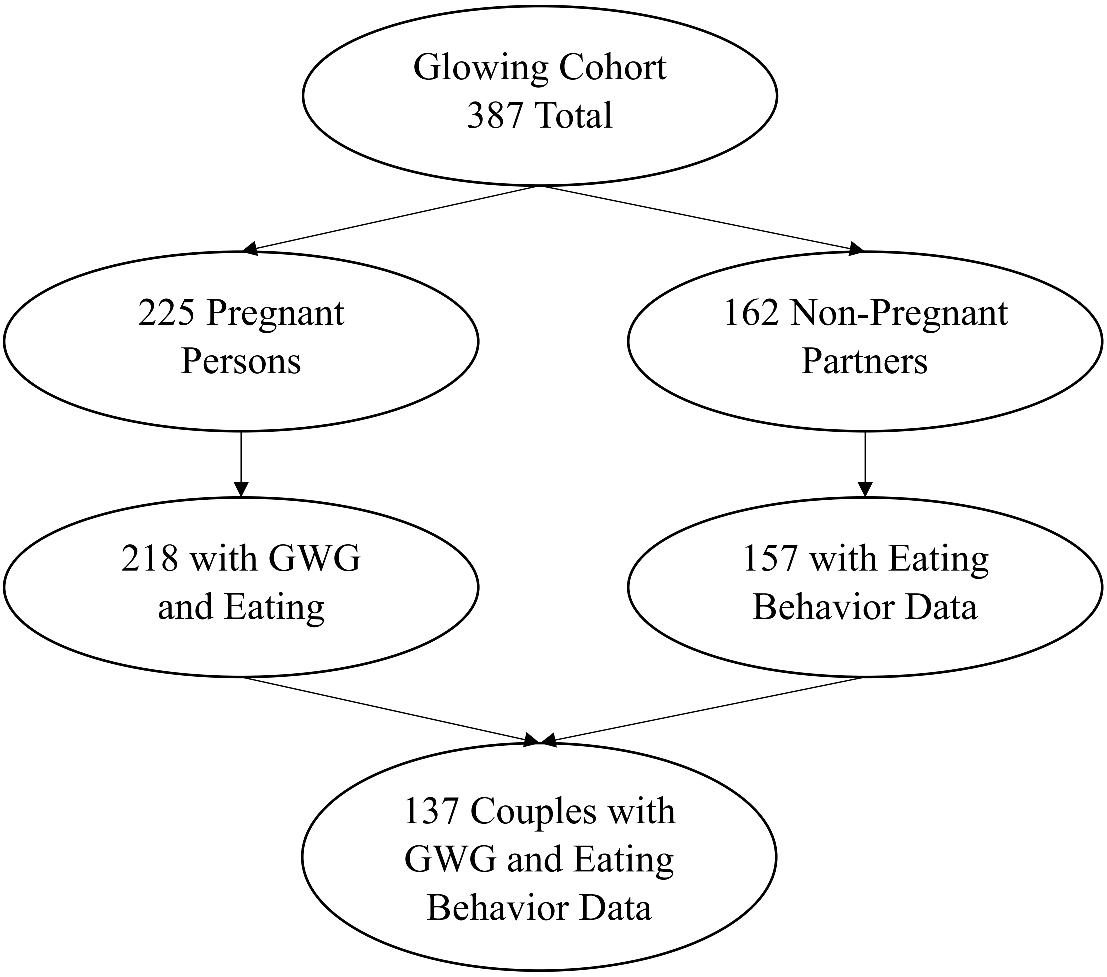

**Table S1. Baseline Characteristics of Non-Pregnant Partner by Enrollment**			
	**Pregnancy (n=131)**	**Postpartum (n=26)**	**p-value**
**Age (Mean±SD)**	30.8±4.0	33.0±4.5	**0.007**
**Body Mass Index (BMI; kg/m^2; Mean±SD)**	28.5±5.2	28.7±4.5	0.834
Normal Weight, N(%)	35 (26.7)	7 (29.3)	0.954
Overweight, N(%)	54 (40.6)	10 (39.0)	
Obesity, N(%)	42 (32.7)	9 (31.7)	
**Race**			0.284
White/Caucasian, N(%)	117 (89.0)	22 (85.4)	
Black/African American, N(%)	14 (11.0)	3 (12.2)	
Asian or more than One Race, N(%)	0 (0.0)	1 (2.4)	
Unknown or Not Reported, N(%)	0 (0.0)	0 (0.0)	
**Highest Education Completed**			0.178
<College Graduate, N(%)	56 (42.7)	15 (59.0)	
≥College Graduate, N(%)	72 (55.2)	11 (41.0)	
Unknown/Not Reported, N(%)	3 (2.1)	0 (0.0)	
**Individual Income**			0.229
<$40,000 USD, N(%)	42 (31.7)	15 (59.0)	
≥$40,000 USD, N(%)	83 (63.4)	11 (41.0)	
Unknown/Not Reported, N(%)	6 (4.9)	0 (0.0)	
**The Eating Inventory**			
Cognitive Restraint, M±SD	6.6±4.8	7.0±4.5	0.625
Low, N(%)	102 (78.2)	22 (82.9)	0.528
High, N(%)	29 (21.8)	4 (17.1)	
Dietary Disinhibition, M±SD	5.4±3.1	5.0±4.0	0.534
Low, N(%)	113 (86.1)	22 (82.9)	0.625
High, N(%)	18 (13.9)	4 (17.1)	
Perceived Hunger, M±SD	4.8±3.2	4.5±2.9	0.677
Low, N(%)	104 (79.2)	22 (82.9)	0.614
High, N(%)	27 (20.8)	4 (17.1)	
Reported as N (%) if not otherwise noted.			

Table S2. Baseline Characteristics of Pregnant Persons and Non-Pregnant Partners			
	**Pregnant Person (n=137)**	**Non-Pregnant Partner (n=137)**	**p-value**
**Age (Mean±SD)**	30.0±3.7	31.4±4.3	0.07
**Body Mass Index (BMI; kg/m^2; Mean±SD)**	26.1±4.3	28.5±5.3	<0.0001
Normal Weight, N(%)	62 (45.3)	39 (28.5)	
Overweight, N(%)	44 (32.1)	55 (40.1)	
Obesity, N(%)	31 (22.6)	43 (31.4)	
**Marital Status***			
Married or Cohabiting, N(%)	125 (91.2)	-	
Single, Divorced, or Unknown/Not Reported, N(%)	12 (8.8)	-	
**Race**			0.70
White/Caucasian, N(%)	112 (81.7)	110 (80.3)	
Black/African American, N(%)	12 (8.8)	16 (11.7)	
Asian or more than One Race, N(%)	2 (1.5)	0 (0.0)	
Unknown or Not Reported, N(%)	11 (8.0)	11 (8.0)	
**Highest Education Completed**			<0.01
<College Graduate, N(%)	46 (33.6)	54 (39.4)	
≥College Graduate, N(%)	81 (59.1)	64 (46.7)	
Unknown/Not Reported, N(%)	10 (7.3)	19 (13.9)	
**Individual Income**			<0.01
<$40,000 USD, N(%)	67 (48.9)	42 (30.7)	
≥$40,000 USD, N(%)	40 (29.2)	80 (58.4)	
Unknown/Not Reported, N(%)	30 (21.9)	15 (10.9)	
Reported as N (%) if not otherwise noted. *Marital Status only reported by Pregnant Persons			

Table S3. Gestational Weight Gain and The Eating Inventory Overall and by Body Mass Index				
Pregnant Person	Overall (137)	Normal Weight (62)	Overweight (44)	Obese (31)
**Gestational Weight Gain (Total; kg)**	11.5±4.4	13.1±3.0*	11.4±4.9*	8.6±4.6
Inadequate, N(%)	51 (37.2)	38 (61.3)ǂ	7 (15.9)	6 (19.4)
Recommended, N(%)	62 (45.3)	24 (38.7)ǂ	23 (52.3)	15 (48.4)
Excess, N(%)	24 (17.5)	0 (0.0)ǂ	14 (31.8)	10 (32.3)
**The Eating Inventory**				
Cognitive Restraint, M±SD	9.9±4.7‡	9.0±4.7ǂ	10.5±4.8	10.6±4.3
Low, N(%)	73 (53.3)‡	36 (58.1)ǂ	20 (45.5)	17 (54.8)
High, N(%)	64 (46.7)‡	26 (41.9)ǂ	24 (54.5)	14 (45.2)
Dietary Disinhibition, M±SD	4.6±3.1	3.0±2.1ǂ	5.5±3.0	6.3±3.7
Low, N(%)	122 (89.1)	61 (98.4)ǂ	37 (84.1)	24 (77.4)
High, N(%)	15 (10.9)	1 (1.6)ǂ	15 (15.9)	7 (22.6)
Perceived Hunger, M±SD	4.6±2.6	4.0±2.3ǂ	5.3±2.7	4.9±2.8
Low, N(%)	119 (86.9)	58 (93.6)ǂ	34 (77.3)	27 (87.1)
High, N(%)	18 (13.1)	4 (6.5)ǂ	10 (22.7)	7 (12.9)
Non-Pregnant Partner	Overall (137)	Normal Weight (62)	Overweight (44)	Obese (31)
**The Eating Inventory**				
Cognitive Restraint, M±SD	6.7±4.6‡	6.8±4.8	6.9±4.3	6.2±4.9
Low, N(%)	110 (80.3)‡	51 (82.3)	35 (79.5)	24 (77.4)
High, N(%)	27 (19.7)‡	11 (17.7)	9 (20.5)	7 (22.6)
Dietary Disinhibition, M±SD	5.2±3.4	5.1±3.2	4.8±3.2	5.9±3.9
Low, N(%)	120 (87.6)	56 (90.3)	39 (88.6)	25 (80.7)
High, N(%)	17 (12.4)	6 (9.7)	5 (11.4)	6 (19.3)
Perceived Hunger, M±SD	4.7±3.2	4.3±3.2	4.7±2.9	5.4±3.5
Low, N(%)	111 (81.0)	53 (85.5)	35 (79.5)	23 (74.2)
High, N(%)	29 (19.0)	9 (14.5)	9 (20.5)	8 (25.8)
Couple (Average)	Overall (137)	Normal Weight (62)	Overweight (44)	Obese (31)
**The Eating Inventory**				
Cognitive Restraint, M±SD	8.3±3.4	7.9±3.5	8.7±3.3	8.4±3.2
Low, N(%)	100 (73.0)	48 (77.4)	30 (68.2)	22 (71.0)
High, N(%)	37 (27.0)	14 (22.6)	14 (31.8)	9 (29.0)
Dietary Disinhibition, M±SD	4.9±2.4	4.1±2.0	5.2±2.2	6.1±2.7
Low, N(%)	125 (91.2)	60 (96.8)	40 (90.9)	25 (80.6)
High, N(%)	12 (8.8)	2 (3.2)	4 (9.1)	6 (19.4)
Perceived Hunger, M±SD	4.6±2.0	4.1±1.8	5.0±2.0	5.1±2.3
Low, N(%)	130 (94.9)	60 (96.8)	42 (95.5)	28 (90.3)
High, N(%)	7 (5.1)	2 (3.2)	2 (4.5)	3 (9.7)

Parent average was calculated the average between pregnant person and non-pregnant partner Eating Inventory. *$p<0.05$ for significance compared to those having obesity; ǂ$p<0.05$ for significance compared to those having overweight or obesity; ‡$p<0.05$ for significance compared between maternal and paternal Eating Inventory constructs
